# Synergistic Effects of BaTiO_3_ and MFe_2_O_4_ (M = Mn, Ni, Cu, Zn, and Co) Nanoparticles as Artificial Pinning Centers on the Performance of YBa_2_Cu_3_O_y_ Superconductor

**DOI:** 10.3390/nano14221810

**Published:** 2024-11-12

**Authors:** Amjad S. Dair, Yassine Slimani, Essia Hannachi, Faten Ben Azzouz, Munirah A. Almessiere

**Affiliations:** 1 Nanotechnology Master Program, Institute for Research and Medical Consultations (IRMC), Imam Abdulrahman Bin Faisal University, P.O. Box 1982, Dammam 31441, Saudi Arabia; 2 Department of Biophysics, Institute for Research and Medical Consultations (IRMC), Imam Abdulrahman Bin Faisal University, P.O. Box 1982, Dammam 31441, Saudi Arabia; 3 Independent Researcher, Bizerte 7021, Tunisia; 4 Department of Physics, College of Science, Imam Abdulrahman Bin Faisal University, P.O. Box 1982, Dammam 31441, Saudi Arabia; 5 Basic and Applied Scientific Research Center, Imam Abdulrahman Bin Faisal University, P.O. Box 1982, Dammam 31441, Saudi Arabia

**Keywords:** YBCO superconductor, co-addition, nanomaterials, electrical resistivity, flux pinning

## Abstract

Large-scale superconductor applications necessitate a superconducting matrix with pinning sites (PSs) that immobilize vortices at elevated temperatures and magnetic fields. While previous works focused on the single addition of nanoparticles, the simultaneous inclusion of different nanoparticles into a superconducting matrix can be an effective way to achieve an improved flux pinning capacity. The purpose of this study is to explore the influence of mixed-nanoparticle pinning, with the co-addition of non-magnetic (BaTiO_3_; BT) and various types of magnetic spinel ferrite (MFe_2_O_4_, abbreviated as MFO, where M = Mn, Co, Cu, Zn, and Ni) nanoparticles, on the superconductivity and flux pinning performances of the high-temperature superconductor YBa_2_Cu_3_O_y_ (YBCO). An analysis of X-Ray diffraction (XRD) data of BT–MFe_2_O_4_-co-added YBCO samples showed the formation of an orthorhombic structure with *Pmmm* symmetry. According to electrical resistivity measurements, the emergence of the superconducting state below $T_{c}^{offset}$ (zero-resistivity temperature) was proven for all samples. The highest $T_{c}^{offset}$ value was recorded for the Y-BT-MnFO sample, while the minimum value was obtained for the Y-BT-ZnFO sample. Direct current (DC) magnetization results showed good magnetic flux pinning performance for all the co-added samples compared to the pristine sample but with some discrepancies. At 77 K, the values of the self-critical current density (self-$J_{cm}$) and maximum pinning force ($F_{pmax}$) for the Y-BT-MnFO sample were found to be eight times higher and seventeen times greater than those for the pristine sample, respectively. The results acquired suggested that mixing the BT phase with an appropriate type of spinel ferrite nanoparticles can be a practical solution to the problem of degradation of the critical current density of the YBCO material.

## 1. Introduction

The high-temperature superconductor (HTSC) YBa_2_Cu_3_O_y_ (YBCO) is an important material for diverse applications in electrical power, fault-current limiters, brushless synchronous motors, magnetron sputtering, energy storage flywheels, etc. [1]. The YBCO compound is a tripled perovskite-type ABO_3_ oxygen-deficient superconductor with a transition critical temperature exceeding the liquid nitrogen temperature (77 K). It has a layered structure along the c-axis composed of CuO_2_ planes and CuO chains that serve as charge reservoirs for the planes. The oxygen content of the YBCO material has an important effect on the superconducting properties.

The parent YBCO material exhibits some intrinsic natural defects that can serve as pinning sites (PSs). However, these intrinsic defects are not sufficient, especially when an external magnetic field is applied. In such a situation, the critical current density $J_{c}$ is rapidly decreased, limiting its use for practical applications. In order to extend the range of superconductor applications, the YBCO matrix incorporated with PSs is efficient for immobilizing vortices at both high temperatures and magnetic fields and is highly demanded. One of the most common ways to solve this issue is to introduce artificial PSs and engineer microscopic defects inside the YBCO matrix.

During the past years, nanomaterials have stormed the field of superconductivity and engrossed the attention of scientists working in this field. Nanomaterials and nanostructures have been widely exploited for enhancing the performance of HTSC [2,3,4]. Introducing nanomaterials and nanostructures into HTSC materials can lead to remarkable energy gains. Indeed, materials at the nanoscale can serve as artificial PSs and can generate microscopic defects resulting in a significant improvement in $J_{c}$ at both the self-field and under an applied external magnetic field. It has been reported that the strains caused by artificial PSs have a key role in the vortex pinning mechanism [5]. Through nanomaterial incorporation, additional defects (so-called vortex defect pinning) can be artificially formed as PSs in superconductor materials [6]. The key to defect pinning is pinning the cores of vortices, which requires the extent of the defects to be similar to the coherence length (ξ) of the superconductor to reach an ideal pinning state. Additionally, the amount and type of incorporated nanomaterial are of great interest for achieving good pinning efficiency of YBCO. Many experiments have been performed on several types of non-superconducting nanoparticles, including metals, semiconductors, and insulators, in the YBCO material to improve its superconducting characteristics [7,8,9]. It has been demonstrated that a suitable amount of nano-inclusions with a specific dimension and shape leads to significant enhancement of the values of $J_{c}$ under an external magnetic field [10,11,12].

Among the various nanostructures used as artificial PSs in a superconducting compound, perovskite-based nanomaterials with the general formula ABO_3_ have been proven to be efficient in upgrading the pinning capacity and increasing the critical current density of the material. For instance, J. Díez-Sierra’s group reported the $J_{c}\;$values and pinning characteristics of YBCO films prepared by chemical solution deposition (CSD) and implanted with various types of perovskite-based nanocrystals including BaTiO_3_ (BT), BaHfO_3_ (BHO), SrZrO_3_ (SZO), and BaZrO_3_ (BZO). Their results showed that the nanostructures containing BHO embedded in YBCO performed best with a homogeneous distribution of nanoparticles. The authors also showed an enhanced self-field $J_{c}\;$for YBCO material containing BT nanoparticles, with an obtained value of 4.6 MA·cm^−2^ in comparison with 4.2 MA·cm^−2^ for the virgin sample. BT-added YBCO film showed quicker decay of in-field $J_{c}$ values due to the agglomeration of BT in the host matrix. Nevertheless, it has an even higher critical transition temperature ($T_{c}$) than the virgin film [3]. In another study, A.K. Jha et al. demonstrated an important improvement in both pinning force ($F_{p}$) and $J_{c}$ for YBCO material embedded with 0.04 of BT compared to pristine YBCO [13]. D. Huang et al. reported an improved critical current density in BT-doped YBCO films by low energy (60 Kev) proton irradiation [14]. P. Mandal et al. recommended a potential scenario of good pinning due to nano-BT insulator particles incorporated into bulk YBCO material [9].

On the other hand, magnetic nanoparticles have received special attention from researchers working in the superconductivity field. Magnetic nanoparticle centers differ from non-magnetic ones in that they can be stronger PSs and more efficient for vortex pinning [15]. Because of the direct magnetic interaction between magnetic PSs and vortices, magnetic nanoparticles can overcome the limitations of defect pinning (arising from non-magnetic impurities that can only interact with the vortex cores and thus can be easily weakened by the temperature effect) [16]. Hence, the magnetic PS effect can be more favorable and operative at elevated temperatures, which is advantageous for practical applications of HTSC. Various studies have been reported in the literature on the impact of magnetic nanoparticles on the superconducting and pinning performance of superconducting materials [17,18,19]. In most cases, the performance and flux pinning potential of the final compound are mainly dependent on the magnetization M of the incorporated magnetic phase into the superconductor material [18]. More specifically, magnetic spinel ferrites offer a likelihood of compatibility with superconducting materials and can be efficiently used as artificial PSs, resulting in enhanced flux pinning and material performance. There are some studies in the literature on this topic. The results differ depending on the type and the amount of spinel ferrite used. For instance, S. C. Wimbush et al. [20] examined the effect of CoFe_2_O_4_ on the superconducting performances of YBCO. Their microstructural analysis showed the development of Y(Fe,Co)O_3_ precipitates rather than the CoFe_2_O_4_ phase. The authors showed that the self-field $J_{c}$ was drastically reduced due to the formation of the undesired precipitates. However, the in-field $J_{c}$ behavior of the added sample is of merit. Compared to the self-field value, the in-field $J_{c}$ of the added sample decreased less quickly than that of the pristine sample, and the values of $J_{c}$ in the added sample are roughly 30% higher in the range of 1–6 Tesla [20]. Sahoo et al. showed that $J_{c}$ was reduced with lower concentrations of CoFe_2_O_4_ in YBCO and then increased [21]. Likewise, A. Abo Arais et al. showed decreases in both $T_{c}$ and $J_{c}$ with a low doping content and then increases with increasing Ni_0.5_Zn_0.5_Fe_2_O_4_ nanoparticles in the YBCO material [22].

Although there is a wealth of research on the role of nanomaterials in superconductors, most of them have focused on single additives or shape effects of the same type of nanostructures. A very limited number of works have been devoted to dual additions of different types of nanomaterials. The mutual interactions of the double addition of nanomaterials can be interesting. Research combining non-magnetic and magnetic nanoparticles and studying their synergistic effects is scarce and needs special interest and more exploration. Hence, in this study, we focus on exploring and studying the role of co-additions of two types of nanomaterials that possess different properties. We selected BT nanoparticles as a non-magnetic PS source for vortex core defect pinning and five different kinds of magnetic spinel ferrites (MFe_2_O_4_, abbreviated as MFO, where M = Mn, Cu, Zn, Ni, and Co) as sources of magnetic PSs. From this standpoint, the performance of YBCO enriched with pre-formed BaTiO_3_ and MFe_2_O_4_ nanoparticles was explored.

## 2. Experimental Details

### 2.1. Synthesis

Pristine YBCO and a set of five different BT–MFe_2_O_4_-co-added YBCO samples were prepared using the solid-state reaction route (SSRR). First, raw materials (supplied from Sigma Aldrich, Burlington, MA, USA) of yttrium oxide (Y_2_O_3_, 99.9%), barium carbonate (BaCO_3_, 99.9%), and copper oxide (CuO, 99.9%) were used to prepare YBCO precursor. The initial ingredients were mixed in a molar ratio of Y:1, Ba:2, and Cu:3 using an agate mortar and pestle. Following grinding and mixing, 750 MPa of pressure was applied to a hydraulic press to turn the resulting mixed powders into pellets. The resulting pellets were heat-treated in a chamber furnace for 12 h at a temperature of 950 °C after being placed into alumina crucibles. After calcination, the pellets were cooled slowly inside the furnace until the ambient temperature was reached. The obtained black pellets were ground to obtain a fine powder of YBCO precursor. During the second step of heat treatment, barium titanate “BT” and magnetic spinel ferrite “MFO” nanoparticles prepared separately by sol gel auto-combustion and hydrothermal routes, respectively, were used as additives. Details of the nanoparticles’ preparation are provided in the Supplementary Materials. Similar amounts of BT (mean size 36 nm) and MFO (mean size range of 10–30 nm) nanoparticles were added simultaneously to the pre-formed YBCO precursor. The amount of the co-additives was 0.1 wt.% for the total mass of the YBCO precursor. The powders for each set were finely mixed and ground using an agate mortar and pestle for approximately half an hour until a homogeneous mixture was obtained. Each powder was then pressed into the shape of a pellet. The prepared pellets were sintered for 8 h at a high temperature of 950 °C and then left to gradually cool down to room temperature. The reference pristine YBCO sample (i.e., without BT and MFe_2_O_4_ co-addition) was re-ground and created under identical conditions as the co-added samples for the purpose of comparison. In the rest of the text of this paper, the samples are labeled and coded as “pristine” for the YBCO sample without co-addition and “Y-BT-MnFO”, “Y-BT-CuFO”, “Y-BT-CoFO”, “Y-BT-NiFO”, and “Y-BT-ZnFO” for the YBCO samples with co-added BT and MnFe_2_O_4_, BT and CuFe_2_O_4_, BT and CoFe_2_O_4_, BT and NiFe_2_O_4_, and BT and ZnFe_2_O_4_, respectively.

### 2.2. Characterization

The crystalline structure and phase formation of different samples were identified using the X-Ray diffraction technique (XRD, Rigaku MiniFlex 600, Tokyo, Japan). The surface morphology and chemical compositions of the samples were analyzed using scanning electron microscopy (SEM, model: Zeiss Merlin, Carl Zeiss SMT, Oberhochen, Germany). The electrical resistivity measurements were performed on rectangular-shaped bars by means of the four-probe method using a Quantum Design magnetic property measurement system (MPMS 3). The DC magnetization measurements were performed under a variable applied magnetic field ranging from −6 to +6 Tesla at different temperatures of 77 K and below using MPMS 3 system. From the obtained M(H) loops and based on the expression of Bean’s extended model, critical current density ($J_{cm}$) values were determined as in [23]:

(1) $$J_{cm}=\frac{20\;\Delta M}{x\left(1-\frac{x}{3y}\right)}\;$$

where x and y are the dimensions of the samples, and ΔM is the width of the magnetization loops.

## 3. Results and Discussion

### 3.1. XRD and SEM Analyses

**Figure 1:**
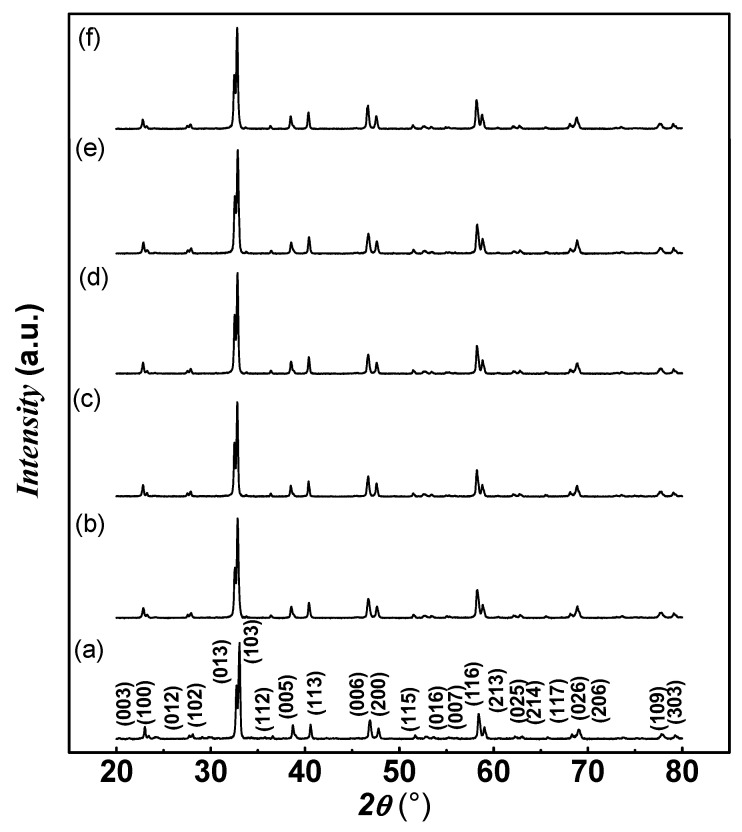
XRD patterns of the (**a**) pristine, (**b**) Y-BT-MnFO, (**c**) Y-BT-CuFO, (**d**) Y-BT-CoFO, (**e**) Y-BT-NiFO, and (**f**) Y-BT-ZnFO samples.

Figure 1 depicts the powder XRD patterns of the pristine, Y-BT-MnFO, Y-BT-CuFO, Y-BT-CoFO, Y-BT-NiFO, and Y-BT-ZnFO samples. The scanned angular range extended from 20° to 80°. The analysis of pristine sample data indicated a single-phase orthorhombic YBCO structure with *Pmmm* symmetry. No peaks relevant to BaTiO_3_ or MFe_2_O_4_ nanoparticles or secondary phases containing Ba, Ti, M (Mn, Co, Cu, Ni, Zn), and Fe elements were noticed under the accuracy of the XRD technique. All the prepared co-added samples remained under orthorhombic symmetry crystallization, and no transition to another symmetry (e.g., tetragonal phase) was observed. The split peaks observed at (021)/(102), (013)/(103), and (026)/(206) provide strong evidence for the formation of an orthorhombic structure for all samples and are also a clear sign of the successful formation of superconducting materials. The lattice parameters for the samples were refined using Match 3! Software (version 3.12 Build 208, CRYSTAL IMPACT, Bonn, Germany) such that the calculated patterns fit the observed spectra very well. The values of lattice parameters (a, b, and c) and the oxygen content y are listed in Table 1. The oxygen content is linked to the parameter c by the following empirical relationship [11]:

(2) y=75.25−5.856 c 

The variation in the lattice parameter c was accompanied by an increase in the oxygen content for the Y-BT-MnFO, Y-BT-CuFO, and Y-BT-CoFO samples, followed by a decrease for the Y-BT-NiFO and Y-BT-ZnFO samples. It is commonly reported that the value of the oxygen content plays a key role in the origin of superconductivity in HTSC materials. The higher oxygen content may be responsible for good interlayer exchange and, therefore, better superconducting properties [11].

**Figure 2:**
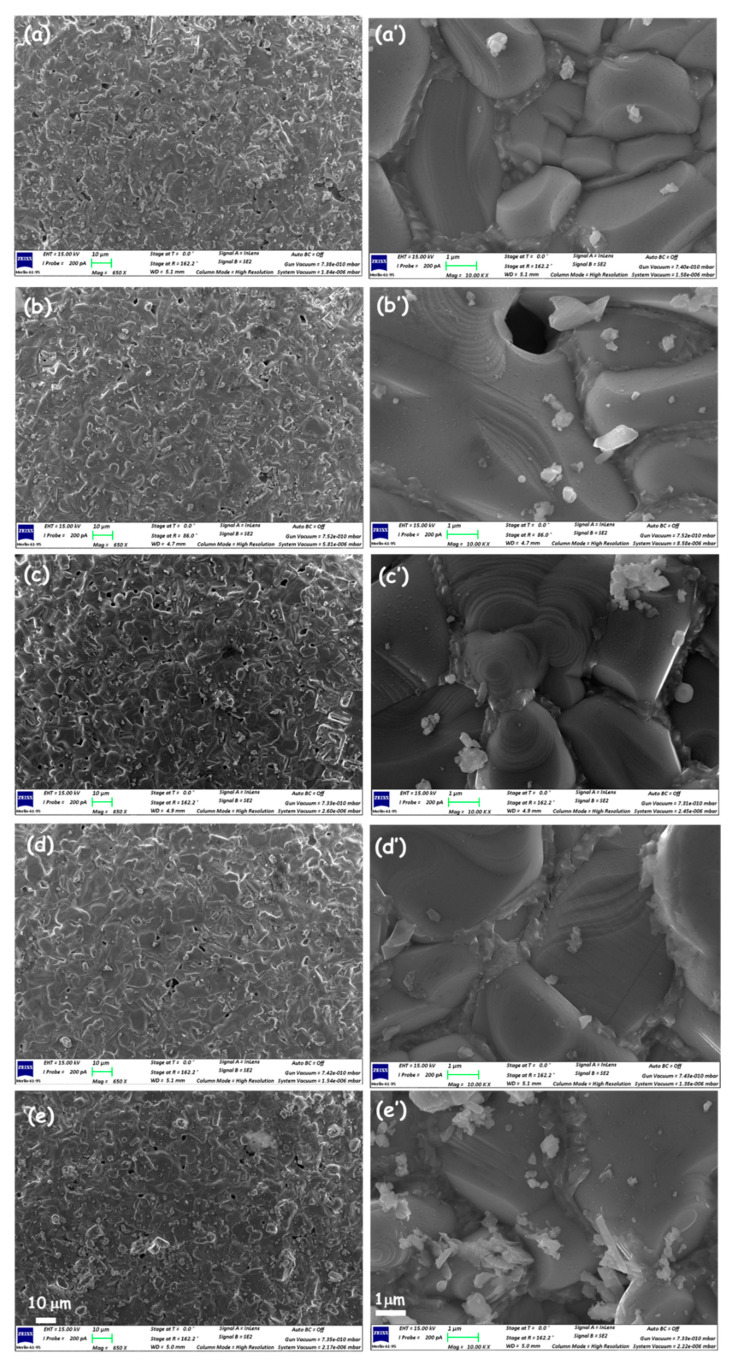
Low-magnification (**left**; scale bar 10 μm) and high-magnification (**right**; scale bar 1 μm) SEM images showing the surface morphologies of the (**a**,**a’**) Y-BT-MnFO, (**b**,**b’**) Y-BT-CuFO, (**c**,**c’**) Y-BT-CoFO, (**d**,**d’**) Y-BT-NiFO, and (**e**,**e’**) Y-BT-ZnFO samples.

SEM observations of the prepared co-added samples Y-BT-MnFO, Y-BT-CuFO, Y-BT-CoFO, Y-BT-NiFO, and Y-BT-ZnFO were performed, and the surface morphologies of the different samples are shown in Figure 2. Low-magnification SEM images (Figure 2a–e) show a granular structure with large grains randomly oriented in different directions, which is a distinctive structure of HTSC materials.

SEM observations under high magnification were also carried out, and the observed images for the Y-BT-MnFO, Y-BT-CuFO, Y-BT-CoFO, Y-BT-NiFO, and Y-BT-ZnFO samples are shown in Figure 2a’–e’, respectively. For each co-added sample, nano-spots were observed on the surfaces of superconductor grains. In addition, the grains appeared to be welded together by clusters and agglomerates that serve as bonds between the grains. A previous report showed that the BT phase favors being positioned between the superconducting grains behaving as a catalyst to improve the quality of the grains’ boundary composition [13]. Then, it can be assumed that the different MFe_2_O_4_ nanoparticles can preferentially spread on the surface of the YBCO matrix rather than in the intergranular regions, where they appear as nano-spots.

### 3.2. Electrical Property Analysis

**Figure 3:**
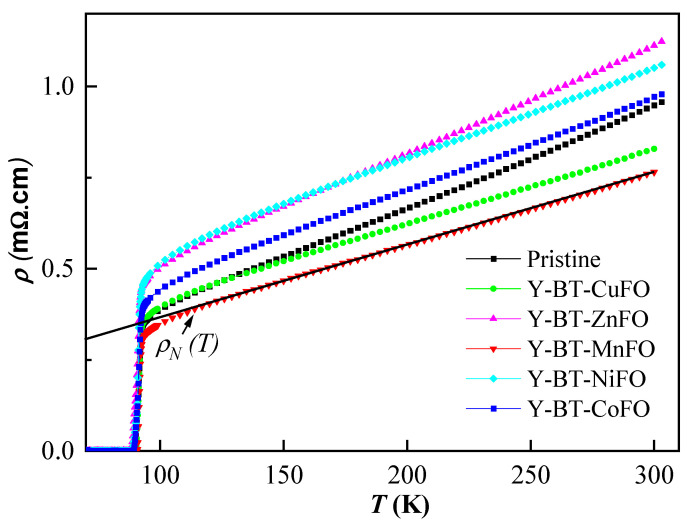
Temperature dependences of electrical resistivity (*ρ*) for the pristine YBCO, Y-BT-MnFO, Y-BT-CuFO, Y-BT-CoFO, Y-BT-NiFO, and Y-BT-ZnFO samples.

For superconducting materials, the interpretation of the electrical resistivity transition versus temperature $\left(\rho (T)\right)$ is valuable for monitoring the percolation conduction mechanism between the grain boundary regions and the superconducting grains. Figure 3 depicts the temperature dependences of electrical resistivity $\left(\rho (T)\right)$ for the pristine YBCO, Y-BT-MnFO, Y-BT-CuFO, Y-BT-CoFO, Y-BT-NiFO, and Y-BT-ZnFO samples.

All curves follow the linear metallic-like behavior (i.e., $\frac{d\rho }{dT}>0$) at high temperatures. In this range, the temperature dependences of the resistivity follow the Anderson and Zou relation [24,25]:

(3) $$\rho_{N}\left(T\right)=\rho_{R}+AT$$

$\rho_{R}$ is the residual resistivity, and A is the resistivity slope, which depends on the intrinsic electronic interactions [26]. The absolute resistivity in the normal state can be influenced by several parameters such as porosity, diffusion of grain boundaries, etc., and its linearity over a large temperature range indicates that the preparation procedure for the samples was executed correctly. The linear behavior is followed by a non-linear region (marked rounding) and a jump in *ρ* corresponding to a transition to the non-ohmic region (superconducting state) in HTSC grains. Compared to that for the pristine sample, the room-temperature resistivity ($\rho_{300K}$) decreased for the Y-BT-MnFO and Y-BT-CuFO samples but increased for the rest of the samples. This suggests that the type of co-additives plays a crucial role in controlling the electrical transport properties of YBCO materials. Further, from the linear plots (indicated by straight lines in the figure), the extrapolation towards 0 K yields the residual resistivity $\rho_{R}$. $\rho_{R}$ is temperature-independent and can be expressed as $\rho_{R}=m^{*}/ne^{2}\tau_{0}$, where $m^{*}$ is the effective mass of electrons and $\tau_{0}$ is the scattering diffusion time resulting from impurities. The values of $\rho_{300K}$ and $\rho_{R}$ in different samples are listed in Table 2. $\rho_{R}$ was higher for the co-added samples than for the pristine one. $\rho_{R}$ is a marker of the sample homogeneity and imperfection density. Among all samples, Y-BT-NiFO had the highest $\rho_{R}$, and its value increased by approximately 2.5 orders of magnitude relative to that of the pristine sample.

**Figure 4:**
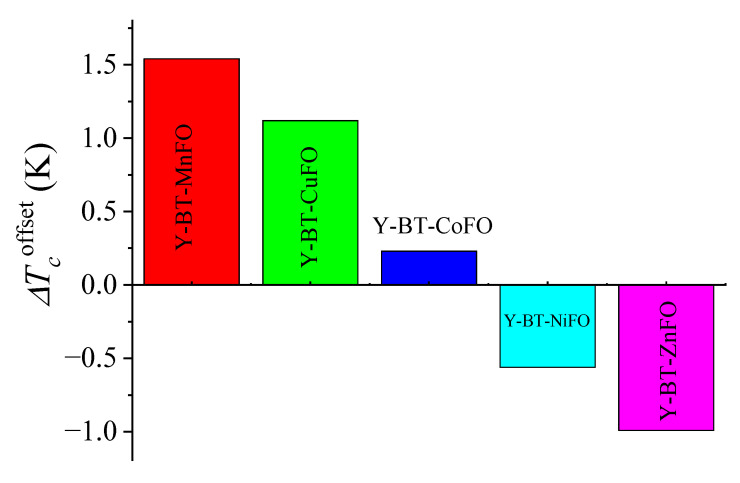
Values of ${\Delta T}_{c}^{offset}$ for the Y-BT-MnFO, Y-BT-CuFO, Y-BT-CoFO, Y-BT-NiFO, and Y-BT-ZnFO samples relative to the pristine sample.

This indicates lower time relaxation due to a higher density of imperfections and inhomogeneities in this sample compared to all as-prepared ones [11]. The notable rounding observed in all samples corresponds to the emergence of fluctuation-induced conductivity. Some pairs of electrons begin to form when the resistivity deviates from linearity below a certain temperature [26]. As the temperature diminishes, the number of electron pairs formed intensifies until the critical mean-field temperature is attained, at which all conducting electrons are coupled and acting in concert. Through the relationship between temperature and resistivity, the zero-resistivity temperature $T_{c}^{offset}$ and the onset transition temperature $T_{c}^{onset}$ can be obtained (Table 2). $T_{c}^{offset}$ is the temperature at which the resistivity has just completely fallen to zero and can be defined as the onset of the global superconductivity temperature of the sample. $T_{c}^{onset\;}$is the temperature at which the ρ−T curves deviate from linear behavior. The $T_{c}^{offset}$ and $T_{c}^{onset}$ values of the pristine sample were ~88.8 K and 93.2 K, respectively, and the transition width $(T_{c}^{onset}-T_{c}^{offset}$) was ~4.4 K. From Table 2, it can be observed that the values of $T_{c}^{onset}$ are virtually constant for all as-prepared samples. However, a notable change in $T_{c}^{offset}$ values is noticed. The transition widths were 2.7 K, 3.0 K, 4.1 K, 4.8 K, and 4.9 K for the Y-BT-MnFO, Y-BT-CuFO, Y-BT-CoFO, Y-BT-NiFO, and Y-BT-ZnFO samples, respectively. The transition width determines the purity of the sample and the quality of the superconducting transition. Hence, the expansion in the transition width is related to the increase in disorder and inhomogeneities in the samples. Compared to the pristine sample, the Y-BT-NiFO and Y-BT-ZnFO samples had wider transition widths. From a crystallographic point of view, several previous reports showed that zinc-doped YBCO showed a sharp decrease in the critical transition temperature without significantly affecting the orthorhombic symmetry of the crystal structure [27], which is consistent with the results obtained in this research. Figure 4 illustrates the ${\Delta T}_{c}^{offset}$ of the Y-BT-MnFO, Y-BT-CuFO, Y-BT-CoFO, Y-BT-NiFO, and Y-BT-ZnFO samples relative to the pristine sample. From the chart, we can clearly confirm that the combination of BT nanoparticles with either NiFO or ZnFO lowered the $T_{c}^{offset}$ of YBCO (${\Delta T}_{c}^{offset}<0$). The rate of $T_{c}^{offset}$ depression was much lower for the Y-BT-NiFO sample. Nevertheless, the co-addition of BT-MnFO, BT-CuFO, and BT-CoFO to the YBCO matrix increased $T_{c}^{offset}$ (${\Delta T}_{c}^{offset}$ > 0). Remarkably, among all as-prepared samples, the Y-BT-MnFO sample had the highest${\;T}_{c}^{offset}$ value.

This indicates that the combined incorporation of BaTiO_3_ and MnFe_2_O_4_ nanoparticles into a YBCO matrix has a positive impact on achieving better superconducting characteristics. This result agrees well with those previously reported by Y.S. Rammah et al. [18]. The authors reported a comparative investigation on the effect of nano-metal oxides of Mn_3_O_4_, Cr_2_O_3_, Co_3_O_4_, SnO_2_, and CuO on the superconducting properties of bulk YBCO. According to their findings, the superconducting transition temperature showed an increase with Mn_3_O_4_ addition and a reduction when doping with other metal oxides. The observed degradation of ${\;T}_{c}^{offset}$ with BT-NiFO or BT-ZnFO co-addition could also be due to a non-uniform distribution of the co-additives inside the superconducting matrix, trapping of the mobile holes, or some other mechanisms associated with oxygen vacancy disturbance [18]. It is commonly known that the concentrations of hole carriers (P) in CuO_2_ planes can influence the superconducting characteristics of a material, mainly the critical transition temperatures. P was calculated from the electrical resistivity using the following expression [28]:

(4) $$P=0.16-{\left[\frac{\left(1-\frac{T_{c}^{offset}}{T_{cmax}}\right)}{82.6}\right]}^{0.5}$$

where $T_{cmax}$ is taken as 92 K for the YBCO superconducting phase [28]. The values of P for different samples are shown in Table 1.  P varied between 0.136 to 0.145, which is consistent with the range value obtained for the YBCO compound [28]. The values of P increased from 0.139 for the pristine sample to 0.145 for the Y-BT-MnFO sample but decreased to reach a minimum value for the Y-BT-ZnFO sample. This infers that the co-addition of BT and MnFe_2_O_4_ nanoparticles had a positive effect on the concentrations of hole carriers, which is consistent with the results showing enhancement of $T_{c}^{offset}$. It appears that the combination of BT and MnFe_2_O_4_ nano-inclusions incorporated into YBCO enhances the quality of grain boundaries, facilitates the percolation of the current, and promotes the electrical transport properties of the material. In contrast, the minimum values obtained specifically for the Y-BT-ZnFO and Y-BT-NiFO samples may be related to a large number of grain boundaries with a low density of charge carriers and consequently smaller $T_{c}^{offset}$ values compared to those in the inner area of superconducting grains.

### 3.3. Magnetic Hysteresis Loop Analysis

**Figure 5:**
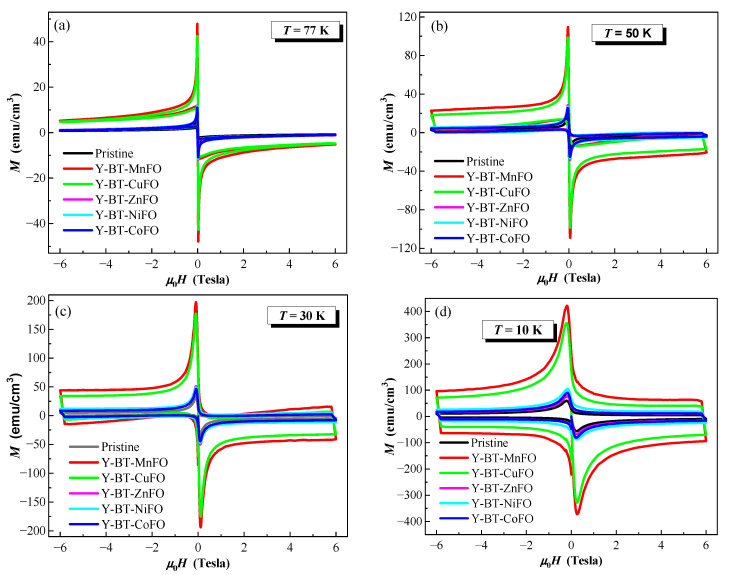
Magnetization hysteresis loops, M(H), for the pristine, Y-BT-MnFO, Y-BT-CuFO, Y-BT-NiFO, Y-BT-CoFO, and Y-BT-ZnFO samples recorded at temperatures of: (**a**) 77 K, (**b**) 50 K, (**c**) 30 K, and (**d**) 10 K.

Figure 5 displays the variations of magnetization M with the magnetic field, M(H) hysteresis loops, the pristine, Y-BT-MnFO, Y-BT-CuFO, Y-BT-NiFO, Y-BT-CoFO, and Y-BT-ZnFO samples recorded in the magnetic field, and $\mu_{0}H$, which ranged from −6 Tesla to +6 Tesla, at various temperatures of 77 K, 50 K, 30 K, and 10 K. Anderson and Kim [29] considered the vortices (i.e., flux lines) in the mixed state of a type II superconductor as specific elastic objects that can be pinned by different structural imperfections, such as dislocations, impurities, strain, etc. This leads to irreversibility in the M(H) hysteresis loops [30].

In addition, $M\left(H\right)\;$loops showed linear dependences at low magnetic fields, reflecting diamagnetic behavior. The peaks observed in *M(H)* loops correspond to the onset penetration of the magnetic field. Of all samples, the Y-BT-MnFO and Y-BT-CuFO samples showed a slight shift in these peaks toward higher fields, suggesting good flux pinning properties in these two systems. For each sample and in a fixed magnetic field, when the temperature decreased, the distance between the lower ($M^{-}(H)$) and upper ($M^{+}(H$)) branches of the M(H) loops (i.e., ΔM) increased. This is due to a decrease in thermal fluctuations, which leads to suppression of the vortex movement as the temperature decreases. On the other hand, by comparing the widths of M(H) loops for all samples at a given temperature, one can notice that the Y-BT-MnFO and Y-BT-CuFO samples showed wider ΔM values than the pristine, Y-BT-NiFO, Y-BT-CoFO, and Y-BT-ZnFO samples. The larger width of ΔM for the Y-BT-MnFO and Y-BT-CuFO samples indicates that these samples contained effective structural defects that contribute to delaying the vortices’ motion, thus improving $J_{c}$ values. These results are in line with the electrical transport analysis. To further validate the synergistic effects of BT and MFO nanoparticles on the flux pinning efficiency, the critical current density $J_{cm}$ was determined from the widths of the M(H) loops using the Bean model expression (Equation (1)).

### 3.4. Self- and In-Field Critical Current Density

**Figure 6:**
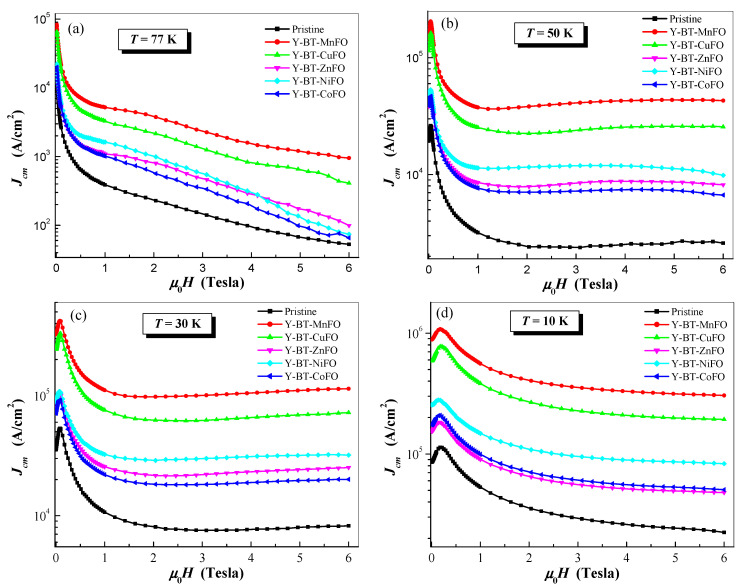
In-field $J_{cm}$ variations for the pristine, Y-BT-MnFO, Y-BT-CuFO, Y-BT-NiFO, Y-BT-CoFO, and Y-BT-ZnFO samples recorded at temperatures of: (**a**) 77 K, (**b**) 50 K, (**c**) 30 K, and (**d**) 10 K.

Figure 6 depicts the magnetic field dependences of $J_{cm}$ at variable temperatures of 77 K, 50 K, 30 K, and 10 K. All co-doped samples showed an improvement in $J_{cm}$ values in the entire applied magnetic field.

**Figure 7:**
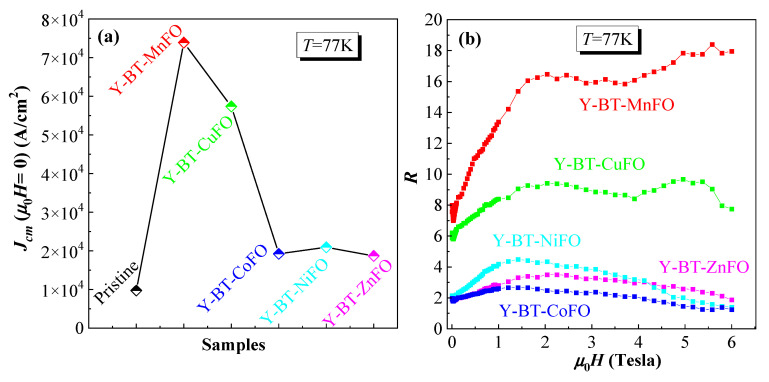
(**a**) Self-field $J_{cm}$ values. (**b**) Variations of the ratio R vs. $\mu_{0}H$.

More interestingly, the Y-BT-MnFO sample showed the highest values of $J_{cm}$ compared to the other samples, indicating better flux pinning efficiency of this sample at operating temperatures between 77 and 10 K. This result is attributed to the formation of a proper density of flux pinning sites due to non-superconducting BT and MnFO nano-inclusions. The values of the self-field $J_{cm}$ at T = 77 K for different samples are illustrated in Figure 7a.

The self-field $J_{cm}$ varied depending on the type of co-additives embedded in the YBCO compound. At 77 K, $J_{cm}$ tracked in the following order: $J_{cm}$(pristine sample) < $J_{cm}$(Y-BT-ZnFO) < $J_{cm}$(Y-BT-CoFO) < $J_{cm}$(Y-BT-NiFO) < $J_{cm}$(Y-BT-CuFO) < $J_{cm}$(Y-BT-MnFO). A maximum value of 7.38 × 10^4^ A/cm^2^ was reached for the Y-BT-MnFO sample, which was eight times higher than that of the pristine sample. The significant increase in self-field $J_{cm}$ for the co-added samples indicates a strong pinning property in these samples. This enhanced pinning property can be attributed to the formation and contributions of effective defects; the contribution of core vortex defect pinning of the order of coherence length (ξ), and the magnetic defect pinning contribution that can directly interact with the flux of the vortex. In addition, it is worth noting that among all the co-added samples, the Y-BT-MnFO and Y-BT-CuFO samples had the highest $J_{cm}$ values. Several possible reasons can be responsible for this result. First, among all the co-added samples, the Y-BT-MnFO and Y-BT-CuFO samples had the highest values for hole carrier concentrations *P* and critical transition temperature $T_{c}^{offset}$. This means that the combination of BT and MFe_2_O_4_ (M = Mn, Cu) nanoparticles in YBCO enhances the grain boundary quality, facilitates current percolation, and ultimately enhances the flux pinning properties of the material. In addition, the Y-BT-MnFO and Y-BT-CuFO samples presented higher oxygen contents, which could also be responsible for the good interlayer exchange and ultimately better superconducting properties. Finally, when comparing the Y-BT-MnFO and Y-BT-CuFO samples, the former presented the best performance. Since both samples contained BT nanoparticles, we can attribute this distinction to the type of magnetic defects contributing to vortex pinning. Therefore, another plausible reason can be assumed, which is mainly related to the type of magnetic nanoparticles. The MnFe_2_O_4_ nanoparticles are classified as mixed spinel ferrites containing Mn^2+^ element, which has the highest magnetic moment (5.9 μ_B_) [18], leading to high magnetization. This high magnetization seems to have an important role in enhancing the strength and contribution of magnetic defects leading to an overall strong vortex pinning mechanism. Thus, a mixed landscape of two types of competitive and effective defects can be assumed to be responsible for the large enhancement observed for the self-field $J_{cm}$ for the Y-BT-MnFO sample: the core vortex defects of a similar size to the coherence length and the magnetic type defects; both of them are caused by the co-addition of BT and MnFO nanoparticles to YBCO. On the other hand, the self-field $J_{cm}$ value was lowest for the Y-BT-ZnFO sample but remained higher compared to that of the pristine sample. The observed lower self-field $J_{cm}$ compared to those of all the prepared co-added samples can be attributed to ZnFO spinel ferrite itself, which includes a non-magnetic Zn^2+^ element (the magnetic moment of Zn^2+^ is zero), which can ultimately lead to the occurrence of weak magnetic defects compared to those in other samples.

To further quantify the role of the BT and the various spinel ferrite co-additions on the improvement in critical current density in the present samples, we compared the in-field $J_{cm}\;$values of the co-added samples with those of the pristine one by calculating the ratio $R=J_{cm}$(co-added sample)$/J_{cm}$(pristine sample). The variations in R ratios versus the applied magnetic field for all co-added samples are plotted in Figure 7b. In the entire considered magnetic field range, all co-added samples had values of R superior to 1, signifying the positive effects of both BT and spinel ferrite nanoparticle co-addition on the flux pinning property. The largest R values were obtained in the Y-BT-MnFO and Y-BT-CuFO samples, with the advantage being evident for the Y-BT-MnFO sample. This means that the mixture of BT and MnFe_2_O_4_ in the YBCO compound was more beneficial and operative than the mixture of BT and CuFe_2_O_4_. At 77 K, the value of critical current density for the Y-BT-MnFO sample was eight-fold greater than that for the pristine sample in a self-magnetic field, and this boosting was continuous and has been found to be up to sixteen-fold for an applied magnetic field of approximately 2–3 Tesla. This result confirms once again that the mixed landscape containing BT and MnFe_2_O_4_ nanoparticles embedded in the YBCO matrix leads to the formation of well-organized and robust pinning sites capable of pinning a larger number of vortices.

**Figure 8:**
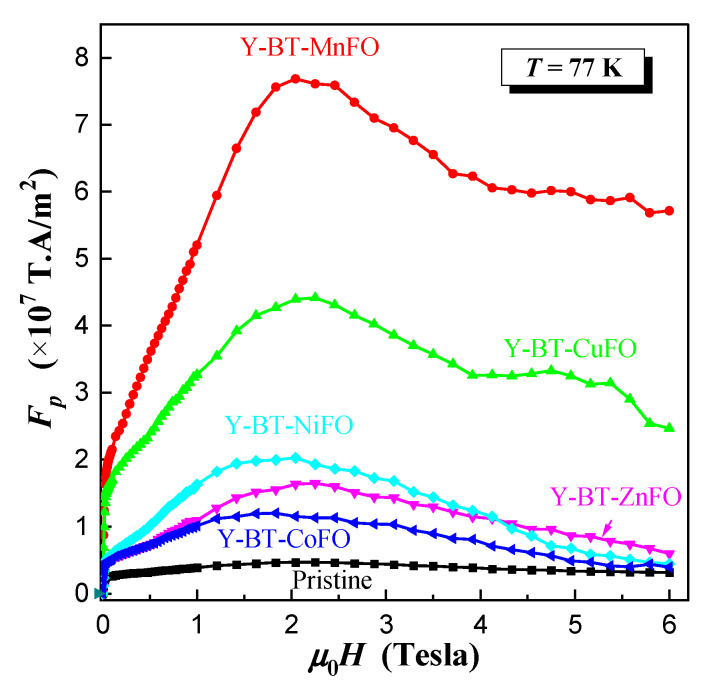
Plots of pinning force density ($F_{p}$) vs. $\mu_{0}H$ for different samples at *T* = 77 K.

To examine the synergistic effects of BT and spinel ferrite nanoparticle co-addition on the flux pinning characteristics, the values of $J_{cm}$ at T = 77 K were used to compute the pinning force $F_{p}=\mu_{0}H\times J_{c}$. Figure 8 displays $F_{p\;}$ against $\mu_{0}H$ at T = 77 K for all prepared samples. The figure shows that the pinning force was significantly increased in the entire applied field for all co-added samples compared to the pristine one. The pinning force increased with increasing magnetic field until reaching a maximum $F_{pmax}\;$ at a certain value of the magnetic field.

Large $F_{p}$ curves obtained for all samples indicate that the pinning property can be interpreted by more than one pinning mechanism in the entire applied magnetic field [31]. The interaction of vortices with a pinning site can arise either from δl, which is associated with the non-superconducting phase implanted in the superconducting matrix leading to the electron mean free path scattering, or δk, which is associated with spatial variation in $T_{c}$ [13,31]. In our case, the mixed landscape of non-magnetic BT nanoparticles and magnetic spinel ferrite could synergistically and competitively enhance the contributions of non-superconducting sites and improve the pinning efficiency either through the interfaces between YBCO/nanoparticles, the defects they generate, or direct magnetic interaction between the flux vortex and the magnetic pinning centers. Among all co-added samples, the Y-BT-MnFO sample showed the highest $F_{pmax}$ value of ~8 × 10^7^ T·A/m^2^, which was 17 times greater than that of the pristine sample. In previous studies, perovskite-based nanoparticles such as the BT phase have been shown to lodge between the superconducting grains behaving as a catalyst to improve the quality of the grains’ boundary composition [13]. This in turn results in an increase in the contact surface among the grains (which subsequently facilitates the flow of charge carriers and enhances conductivity) and ultimately leads to intensification of the density of pinning sites via the formation of active vortex core defects. The spinel ferrite nanoparticles, being magnetic in nature, may serve not only as conventional pinning centers but can also enhance the pinning ability via direct interaction with the flux of vortex, eventually leading to their successful immobilization [32]. Another observation that can be discerned from Figure 7 and Figure 8 is the competitive effects of the co-addition of BT-NiFO and BT-ZnFO. It is worth noting that the Y-BT-NiFO sample was better than the Y-BT-ZnFO sample in the range of an applied magnetic field below 4.5 Tesla. Beyond this value, the Y-BT-ZnFO sample became more effective and even better than the Y-BT-CoFO sample, which presented the lowest overall in-field efficiency among all prepared co-added samples. However, at a low temperature (i.e., 10 K; Figure 6d), it was observed that the in-field $J_{cm}$ for the Y-BT-CoFO sample became slightly better than that of the Y-BT-ZnFO sample in the entire applied magnetic field. This indicates that the Y-BT-ZnFO sample was more operative at 77 K.

**Figure 9:**
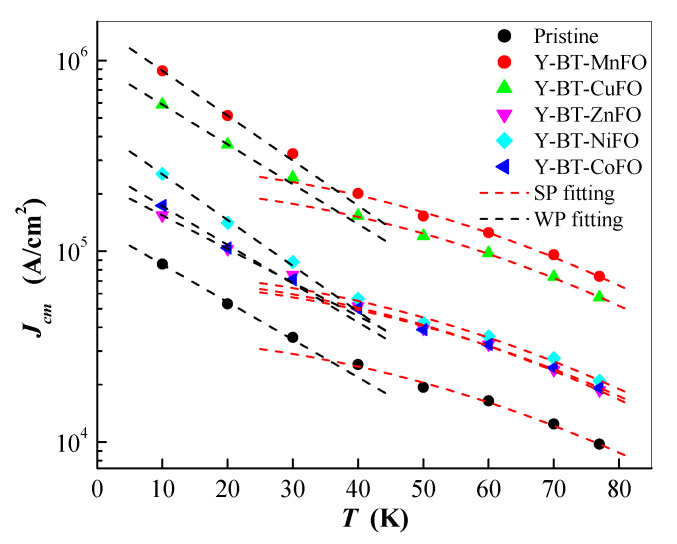
Temperature dependences of $J_{cm}$ for the prepared samples. The dashed lines are the fitting results for the WP (black) and SP (red) contributions.

The temperature dependences of $J_{cm}$ at the self-magnetic field (i.e., 0 Tesla) were plotted, and the results are shown in Figure 9. From these plots, it is possible to categorize the strength of the effective artificial PSs according to their thermal activation process. The potency of pinning can be classified into strong pinning (SP) and weak pinning (WP), and each classification has a typical dependency on the magnetic field and temperature. For the WP and SP categories, the temperature dependences of $J_{cm}$ can be expressed using the following equations, respectively [33,34]:

(5) $$J_{cm}^{WP}=J_{cm}^{WP}\left(0\right)exp(-T/T_{0}^{WP})$$

(6) $$J_{cm}^{SP}=J_{cm}^{SP}\left(0\right)exp(-3{\left(T/T_{0}^{SP}\right)}^{2})$$

where $J_{cm}^{WP}\left(0\right)$ and $J_{cm}^{SP}\left(0\right)\;$are the critical current densities at 0 K for WP and SP contributions, respectively. $T_{0}^{WP}$ and $T_{0}^{SP}$ are characteristic temperatures that determine the pinning energy scale for each contribution. The plots of $J_{cm}$ versus temperature show two regions with two distinct slopes for the pristine and co-added samples, indicating the presence of two flux pinning strengths for each sample. Upon increasing the temperature from 10 K to 77 K, a noticeable drop in $J_{cm}$ was initially observed up to 30 K, and the variation fit well with Equation (5), indicating the dominance of the WP contribution in this temperature regime. Beyond 30 K, a softer decay of $J_{cm}\;$was noticed, and the variations fit well with the SP contribution. A similar tendency has been previously reported in other works [31,35,36]. Generally, SP sites are mostly associated with columnar defects, twins, nano-inclusions with a size in the range of the coherence length $\left(\xi \right)$, and the interfaces between nano-inclusions and the superconducting medium [31]. In contrast, WP sites mostly result from point defects such as atomic substitution and oxygen vacancies [37]. From Figure 9, it can be observed that both the $J_{cm}^{WP}\left(0\right)$ and $J_{cm}^{SP}\left(0\right)$ contributions were altered with the co-addition of nanoparticles inside the YBCO material.

**Figure 10:**
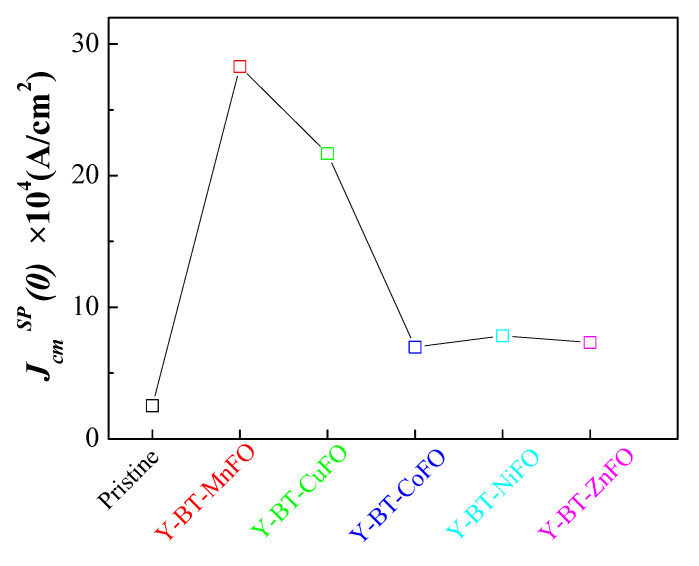
Values of $J_{cm}^{SP}\left(0\right)$ obtained from fitting $J_{cm}\left(T\right)$ dependences at the self-magnetic field for pristine and different co-added samples.

In particular, the$\;J_{cm}^{SP}\left(0\right)$ contribution was directly associated with the presence of nanoparticles. Figure 10 shows the $J_{cm}^{SP}\left(0\right)$ values for different samples. At the considered magnetic field, the value of $J_{cm}^{SP}\left(0\right)$ was higher for all the prepared co-added samples than for the pristine one. More intriguingly, the Y-BT-MnFO sample presented the highest $J_{cm}^{SP}\left(0\right)$ values, while the minimum values were recorded for the Y-BT-ZnFO and Y-BT-CoFO samples. The values of $J_{cm}^{SP}\left(0\right)$ increased by a factor of ~11 for the Y-BT-MnFO sample compared to the pristine sample. This means that the incorporation of BT and MnFO nanoparticles inside the material promoted the number of strong pinning sites in the YBCO material. In light of this comparative study, it can be concluded that the mixed landscape of BT and MnFO nanoparticles in the YBCO superconductor is a successful way to intensify the effective pinning defects and ultimately enhance the superconducting properties of bulk superconducting materials.

We compared our findings for the effects of BT and MFO nanoparticle co-addition with other published results in the literature on other single- and dual-nanoparticle addition (Table 3). The values of $T_{c}^{offset}$ are comparable to those reported in the literature for bulk YBCO material prepared by SSSR [13,21,28,38,39,40,41] and are higher than those of YBCO samples prepared by the sol gel (SG) method [39]. As shown in Table 3, our samples possessed higher values of $J_{cm}$ than those obtained for YBCO doped only with single nanoparticles such as 0.4 wt.% BT phase [13] and WO_3_ nanoparticles [38]. The obtained values are also higher than those recently reported in a YBCO bulk system prepared with dual-nanoparticle addition using SSSR, such as BT/WO_3_ nanoparticles [41], Dy_2_O_3_/WO_3_ nanoparticles [28], and Dy_2_O_3_/Ag nanoparticles [28]. The obtained values of $J_{cm}$ are interesting and look encouraging from the practical standpoint. Yet, these values can be further enhanced by preparing samples in the form of thin films as previously published for other YBCO thin-film systems prepared by CSD [3] or pulsed layer deposition (PLD) [20,42,43] techniques, where the values of $J_{cm}$ can reach 10^6^ at the operating temperature (77 K).

## 4. Conclusions

The combined impacts of BaTiO_3_ (BT) and different types of magnetic spinel ferrites (MFe_2_O_4_ or MFO; M = Mn, Zn, Cu, Ni, and Co) as artificial pinning sites were systematically studied and compared. XRD and electrical transport analyses proved the successful formation of superconducting materials. SEM images showed a granular structure for all samples, with nano-spots dispersed on the surface of grains and clusters and agglomerates present between the grains. The electrical resistivity measurements showed an increase in $T_{c}^{offset}$ and a decrease in the $\rho_{300K}$ for the Y-BT-MnFO sample compared to the pristine sample. The electrical transport results were consistent with the DC magnetization measurements. The analysis showed that the type and the magnetic nature of the additives used have a great impact on the magnetic response of the YBCO compound. Our comparative study showed good magnetic flux pinning performance of all the co-added samples compared to the pristine sample, with some discrepancies. The highest efficiency was observed in the Y-BT-MnFO sample, followed by Y-BT-CuFO sample. A moderate and reasonable increase was also noticed in the Y-BT-NiFO sample, followed by Y-BT-CoFO and Y-BT-ZnFO samples. Particularly, the width of the magnetic hysteresis loops’ ΔM was larger for Y-BT-MnFO, suggesting good flux pinning properties in this material. A noteworthy enhancement in the values of self-field $J_{cm}$, in-field $J_{cm}$, and $F_{p}$ was observed for the Y-BT-MnFO sample compared to the pristine YBCO and the other co-added samples. At 77 K, the self-$J_{cm}$ and $F_{pmax}$ values were eight-fold and seventeen times higher, respectively, for the Y-BT-MnFO sample than those for the pristine sample. These increases were sustained even in the presence of the applied field wherein the improvement of in-field $J_{cm}$ reached up to 16-fold. These results were ascribed to the successful development of a superconducting material of mixed landscapes consisting of non-magnetic BT and magnetic MnFO nanoparticles embedded accurately in the YBCO matrix. The competitive and synergistic effects of both non-magnetic BT and highly magnetic MnFO nanoparticles led to the formation of well-organized vortex core-type defects and flux-magnetic-type defects that can act as strong pinning sites capable of immobilizing a larger number of vortices. The outcomes obtained suggest that the hybrid landscapes with co-addition of BT and MnFe_2_O_4_ nanoparticles can be an efficient strategy and practicable solution to the critical current degradation issue of the bulk YBCO and pave the way for its practical exploitation in transmission energy applications.

## Figures and Tables

**Table 1:** Values of structural parameters for different prepared samples deduced from XRD analysis.

Samples	a (Å)	b (Å)	c (Å)	y
Pristine	3.8196	3.8866	11.6796	6.85
Y-BT-MnFO	3.8190	3.8857	11.6788	6.86
Y-BT-CuFO	3.8151	3.8850	11.6777	6.86
Y-BT-NiFO	3.8188	3.8850	11.6789	6.86
Y-BT-CoFO	3.8205	3.8867	11.6849	6.82
Y-BT-ZnFO	3.8208	3.8872	11.6849	6.82

**Table 2:** Values of superconducting parameters for different prepared samples.

Samples	$T_{c}^{onset}\;(K)$	$T_{c}^{offset}\;(K)$	$\rho_{300K}\;(m\Omega \cdot cm)$	$\rho_{R}\;(m\Omega \cdot cm)$	P
Pristine	93.2	88.8	0.95	0.12	0.139
Y-BT-MnFO	93.1	90.3	0.76	0.15	0.145
Y-BT-CuFO	93.0	89.9	0.83	0.21	0.143
Y-BT-NiFO	93.1	88.2	1.05	0.29	0.137
Y-BT-CoFO	91.1	89.0	0.97	0.21	0.140
Y-BT-ZnFO	92.7	87.8	1.11	0.23	0.136

**Table 3:** Values of $T_{c}^{offset}$ and $J_{cm}$ reported in the current and previously published works.

Samples	$T_{c}^{offset}\;(K)$	$J_{cm}\;(A/{cm}^{2})$	Preparation Method	Ref.
YBCO + 0.4% BTO	~90	~0.3 × 10^4^ (77 K)	SSRR	[13]
YBCO + 1wt.% CoFe_2_O_4_	88.4	1.84 × 10^5^ (40 K)	SSRR	[21]
YBCO + 0.05 wt.% WO_3_ NPs	91.7	0.91 × 10^4^ (77 K)	SSRR	[38]
YBCO + 5wt.% CoFe_2_O_4_	83.15	-	SSRR	[39]
YBCO + 5wt.% CoFe_2_O_4_	73.93	-	Sol-Gel (SG)	[39]
YBCO + 0.1wt.% Co_0.5_Zn_0.5_Fe_2_O_4_	86	-	SSRR	[40]
YBCO + 0.05 wt.% (BTO/WO_3_)	89.65	0.32 × 10^4^ (77 K)	SSRR	[41]
YBCO + (Dy_2_O_3_/WO_3_)	89.7	0.19 × 10^4^ (77 K)	SSRR	[28]
YBCO + (Dy_2_O_3_/Ag)	90.3	0.49 × 10^4^ (77 K)	SSRR	[28]
YBCO + CoFe_2_O_4_ thin film	87	0.25 × 10^6^ (77 K)	PLD	[20]
YBCO + (CoFe_2_O_4_)_0.3_(CeO_2_)_0.7_ multilayer thin film	~90	6.36 × 10^6^ (77 K)	PLD	[42]
YBCO + (La_0.7_Sr_0.3_MnO_3_)_0.5_(CeO_2_)_0.5_ interlayer thin film	90.2	6.56 × 10^6^ (77 K)	PLD	[43]
Pristine YBCO thin film	91.3	4.2 × 10^6^ (77 K)	CSD	[3]
YBCO + BTO thin film	92.3	4.6 × 10^6^ (77 K)	CSD	[3]
YBCO + BZO thin film	90.0	2.36 × 10^6^ (77 K)	PLD	[43]
Pristine YBCO	88.8	0.97 × 10^4^ (77 K)	SSRR	Present work
Y-BT-MnFO	90.3	7.38 × 10^4^ (77 K)	SSRR	Present work
Y-BT-CuFO	89.9	5.73 × 10^4^ (77 K)	SSRR	Present work
Y-BT-CoFO	89.0	1.92 × 10^4^ (77 K)	SSRR	Present work
Y-BT-NiFO	88.2	2.09 × 10^4^ (77 K)	SSRR	Present work
Y-BT-ZnFO	87.8	1.87 × 10^4^ (77 K)	SSRR	Present work

## Data Availability

The raw data supporting the conclusions of this article will be made available by the authors on request.

## Supplementary Materials

The following supporting information can be downloaded at: https://www.mdpi.com/article/10.3390/nano14221810/s1, Heading S1: Synthesis of BaTiO_3_ (BT) nanoparticles. Heading S2: Synthesis of spinel ferrite nanoparticles MFe_2_O_4_ (M = Mn, Cu, Zn, Ni, and Co).
