# Supplementary material for: Synergistic Effects of BaTiO3 and MFe2O4 (M = Mn, Ni, Cu, Zn, and Co) Nanoparticles as Artificial Pinning Centers on the Performance of YBa2Cu3Oy Superconductor
Source: Nanomaterials (Basel). 2024 Nov 12;14(22):1810. doi: 10.3390/nano14221810 (PMC11597904; doi:10.3390/nano14221810)
Supplement: Supplementary file 1 [file nanomaterials-14-01810-s001.zip › nanomaterials-3269406-supplementary.pdf]

nanomaterials

Supplementary Materials

S1. Synthesis of $BaTiO_3$ (BT) nanoparticles

BT nanoparticles were prepared via sol-gel auto-combustion process. Titanium isopropoxide $Ti[OCH(CH_3)_2]_4$ (Merck; purity 98.9%) was dissolved in a mixed solution of of ethanol (Merck) and distilled water, stirred in a water bath at 90 °C for 30 min, and designated as 'solution 1'. Simultaneously, barium acetate (Sigma Aldrich; 99%) powder was mixed in ethanol and distilled water on magnetic stirrer without heating and designated as 'solution 2'. The two solutions 1 and 2 were merged, and stirred by using a magnetic bar at 80 °C for about 15 min to form a homogenous solution. Then, drops of citric acid solution were added as fuel under continuous stirring and heating at 120 °C. The mixed solution was slowly evaporated above the magnetic stirrer until a viscous gel was attained. Then, the temperature was increased, causing the gel to burn due to the self-propagating combustion reaction, and the gel transformed into ashes, which were collected, and then ground to obtain a fine powder. Finally, the resulting powder was calcined for 5 h at 800 °C in a chamber furnace to get the desired nano-$BaTiO_3$ crystalline powder, which was subsequently stored for later use as an additive.

S2. Synthesis of spinel ferrite nanoparticles MFe_2O_4 (M = Mn, Cu, Zn, Ni, and Co)

MFe_2O_4 spinel ferrite nanoparticles were prepared by using hydrothermal route. To do this, appropriate amounts of metal (M= Co^{+2}, Ni^{+2}, Zn^{+2}, Mn^{+2}, Cu^{+2}) nitrates were weighed and dissolved in deionized water under continuous stirring without heating till the solution becomes transparent ('solution 1'). Iron nitrate was also dissolved in distilled water under constant stirring till it was completely dissolved (solution '2'). The two solutions are combined with continuous stirring for approximately 30 minutes. Then, followed by the addition of ammonium hydroxide solution until a pH=7 is reached. The obtained solution was placed into Teflon-lined autoclave chambers and subjected to heat-treatment at 180 °C for 10 h. After the heating process was finished, the autoclave was kept cooled down naturally to ambient temperature. The solid products were gathered, and they were then repeatedly washed in distilled water and ethanol to get rid of the remaining impurities in the sample. The final products were dried and finally, spinel ferrite ($CoFe_2O_4$, $NiFe_2O_4$, $ZnFe_2O_4$, $MnFe_2O_4$, $CuFe_2O_4$) nanoparticles were obtained and stored for later use as additive.
